# Telerehabilitation as a Form of Pulmonary Rehabilitation in Chronic Lung Disease: A Systematic Review

**DOI:** 10.3390/healthcare10091795

**Published:** 2022-09-17

**Authors:** Gregory Reychler, Elise Piraux, Marc Beaumont, Gilles Caty, Giuseppe Liistro

**Affiliations:** 1 Institut de Recherche Expérimentale et Clinique (IREC), Pôle de Pneumologie, ORL et Dermatologie, Université Catholique de Louvain, Avenue Hippocrate 55, 1200 Brussels, Belgium; 2 Service de Pneumologie, Cliniques Universitaires Saint-Luc, Avenue Hippocrate 10, 1200 Brussels, Belgium; 3 Secteur de Kinésithérapie et Ergothérapie, Cliniques Universitaires Saint-Luc, Avenue Hippocrate 10, 1200 Brussels, Belgium; 4 Département de Kinésithérapie, Haute École Léonard de Vinci, Parnasse-ISEI, 1200 Brussels, Belgium; 5 Pulmonary Rehabilitation Unit, Morlaix Hospital Centre, 29600 Morlaix, France; 6 Inserm, University Brest, CHRU Brest, UMR 1304, GETBO, 29200 Brest, France; 7 Service de Médecine Physique, Centre Hospitalier Wallonie Picarde (CHWAPI), 7500 Tournai, Belgium

**Keywords:** chronic lung disease, telerehabilitation, pulmonary rehabilitation, COPD, exercise, fibrosis

## Abstract

Introduction: Tele-rehabilitation is increasingly used to deliver pulmonary rehabilitation. The aim of this systematic review was to compare the effect between tele-pulmonary rehabilitation and classical supervised pulmonary rehabilitation. Method: Three databases were analysed (PubMed, PEDro, Scopus). The selection and evaluation of studies followed the PRISMA guidelines. The risk of bias was evaluated using the PEDro Scale. Results: From the initial selection (*n* = 245), ten studies were retrieved, including from 10 to 67 patients. All but two (IPF) included patients with COPD. Based on the FEV1, patients with COPD were mainly categorised as moderate and severe. The teleactivities were heterogenous in terms of proposed exercises and way of settings and often not in agreement with the guidelines about pulmonary rehabilitation. Despite this, the effects of the interventions were globally positive on functional exercise capacity, quality of life, anxiety and depression, and impact of COPD on personal life but not on dyspnoea. The PEDro scores varied from 4 to 8. The adherence was higher than 80% when supervision during the exercise was included. Conclusion: This review demonstrated that the telerehabilitation is safe and well accepted by the patients, and could be considered as one option of classical pulmonary rehabilitation to improve the functional exercise capacity, quality of life, anxiety and depression, and the impact of COPD on personal’s life. This conclusion cannot be extrapolated to the other chronic lung diseases due to the lack of data.

## 1. Introduction

Pulmonary rehabilitation is an important part of the treatment of all the chronic respiratory diseases even if the benefits and the levels of evidence differ between these diseases [1]. It has been included for a long time in the international guidelines. The main outcomes that were improved were dyspnoea, quality of life, and functional exercise capacity.

Until now, the pulmonary rehabilitation has been mainly provided in specific centres even if it can also be efficiently practised out of these centres as it was more recently highlighted in some studies [2]. Some important barriers related to the pulmonary rehabilitation are retrieved in the literature. The limited access to the centres providing such a program is one of these important barriers [3]. Moreover, heavy financial strain and burden are associated to the cost of the medications and cares. The pulmonary rehabilitation makes an important contribution to them due to the costs associated with the travel, transportation, and parking facilities. Finally, the fixed schedule of the sessions is also a constraint for the patients and relatives [4,5].

Tele-medicine used different technological means to provide a medical approach at home in place of the traditional face-to-face approach of medicine. Tele-rehabilitation is one branch of tele-medicine adapted to the world of the rehabilitation. It tends to maintain the advantages of a classical rehabilitation and to overcome its disadvantages by providing easier access to healthcare services at home with an improved adherence [6]. The feasibility and effectiveness of tele-rehabilitation have previously been demonstrated in many medical conditions [6]. The hypothesis that this could be applied to the pulmonary diseases has to be verified. Summarising knowledge from the literature related to the chronic respiratory diseases is important due to the recent emerging data.

The aim of this systematic review was to summarise the different forms of pulmonary telerehabilitation to highlight its effects and to compare them with a classical supervised pulmonary rehabilitation.

## 2. Material and Methods

### 2.1. Literature Search and Selection

The systematic literature search was based upon the Preferred Reporting Items for Systematic Reviews and Meta-Analyses (PRISMA) statement [7]. It was performed in three databases: PubMed, PEDro, Scopus. The data search was performed from inception to December 2021.

The search strategy used in PubMed was divided based on PICO criteria without including the comparison and outcomes in the equation and using key terms combined with Boolean operators: (a) participant: (suffering from chronic lung disease); (b) intervention: all the forms of telerehabilitation; (c) comparison: usual care or pulmonary rehabilitation; and (d) outcomes: functional exercise capacity, dyspnoea, QoL, and anxiety or depression. The strategy was adapted to the other databases. The lead author (GR) also performed manual tracking of citations in the selected articles.

### 2.2. Inclusion and Exclusion Criteria

The RCTs comparing the effects of telerehabilitation with a classical pulmonary rehabilitation or usual cares in adult subjects diagnosed with a chronic lung disease (bronchiectasis, COPD, asthma…) were included. The definition of the telerehabilitation was the use of at least one technological tool (connected device or any tool offering a visual contact) to follow a home-based program. An isolated phone call to stimulate an active behaviour was empirically considered as not a telerehabilitation tool. Studies had to report at least one of the following parameters as an outcome: functional exercise capacity, dyspnoea, QoL, and anxiety or depression. We included studies published in English or French and with no date restriction. Abstracts, reviews, prospective and retrospective observational cohort studies, editorials, letters and case reports were not included in the systematic review. Studies related to lung cancer or including duplicated data reported in earlier publications were excluded.

### 2.3. Study Selection

Eligible studies were identified and reviewed independently by two reviewers (GR and GL) against selection criteria. After removing duplicates, studies were first screened based on titles and abstracts. Full-text articles were then screened when the title and abstracts were unclear. Discrepancies in selecting studies were resolved by a third independent reviewer (EP).

### 2.4. Data Extraction and Analysis

Characteristics of studies (authors, year, and country), patient data (sex, age, height, weight, and disease), characteristics of the intervention (setting, length, duration per session, frequency, type, and intensity), and outcome data were manually extracted by one qualified reviewer (GR) and presented in tables. The clinical significance thresholds were fixed at 35 m, 1 point, and 4 points for 6MWT, Borg scale, and Saint-Georges Respiratory Questionnaire, respectively. Data presented in graphs were extracted by software tools. The missing data were mentioned as “non-reported” (NR). The collected values were presented as mean ± SD or median (IQR).

### 2.5. Risk of Bias

The methodological quality of the selected studies was assessed using the PEDro scale. This tool contains 11 items with a maximum score of 10. The studies were scored by the lead author (GR) and compared to the PEDro database. Any uncertainty was discussed with a second assessor (EP) until consensus was reached.

## 3. Results

### 3.1. Study Selection

**Figure 1:**
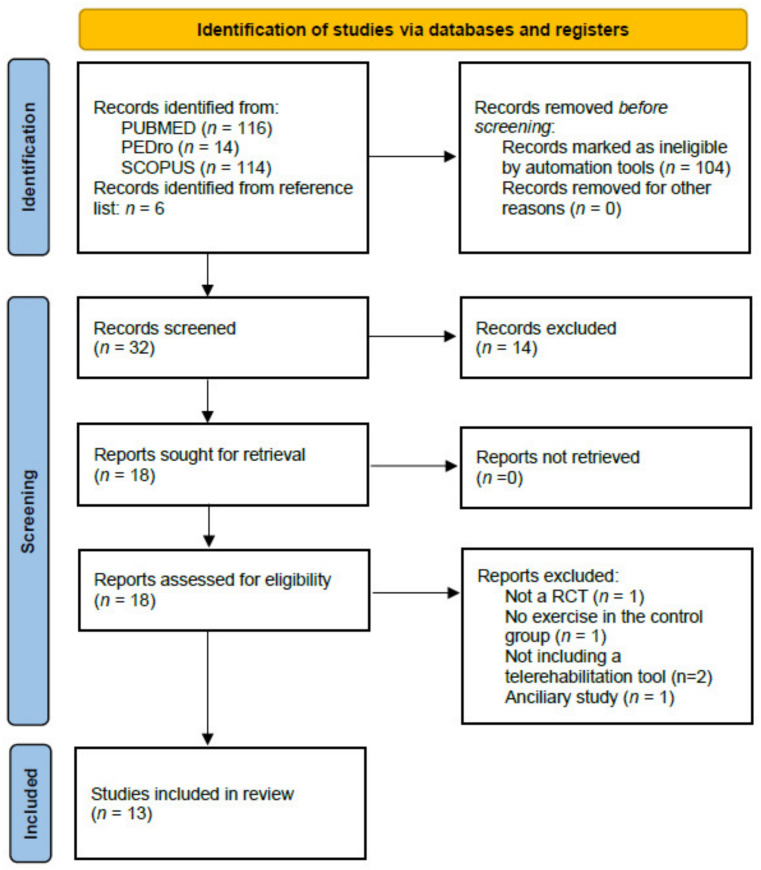
PRISMA flow diagram.

The selection process is highlighted in the PRISMA flow diagram (Figure 1). The search strategy yielded 244 citations from the three databases. Six additional studies were retrieved from manual searching within the reference lists. The full texts of the 18 remaining relevant records were then analysed for eligibility. Out of them, five articles were removed because of not meeting the inclusion criteria. Thirteen studies were retained for the final analysis [8,9,10,11,12,13,14,15,16,17,18,19,20].

### 3.2. Study Characteristics

The characteristics of the studies are reported in Table 1. The analysed studies included one cross-over study [19].

### 3.3. Participants

Participant characteristics are reported in Table 1. The included studies involved 957 participants (10 to 67 by group), of which 486 were in the IG. The mean age of the patients was 67.6 years and the majority of them were men (65%). Two studies included patients with idiopathic pulmonary fibrosis (IPF) [13,18] and all the others included patients with COPD. One study included both heart failure (CHF) and COPD. The mean BMI was higher than 25 in all but one study that reported it. The mean FEV1 was around 50% of predicted value when patients with COPD were included. Based on the mean FEV1 expressed in predicted values, patients included in the studies can be categorised as moderate and severe in 5 and 3 studies, respectively.

### 3.4. Intervention Characteristics

The characteristics of physical telerehabilitation programs are summarised in Table 2A,B. Fifty percent of the studies compared a telerehabilitation program as intervention group (IG) with either usual cares (UC) (Table 2) [8,9,10,11,12,13,14,15,16,17,18,19,20] or with one form of exercises program [8,10,12,15,16,19,20] as control group (CG) (Table 2). One of the studies combined the two comparisons [17]. Two studies focused on the assessment of a maintenance program [17,20]. The teleactivities were heterogeneous in terms of proposed exercises and way of settings. It varied from a simple phone call added to the exercise, to a videoconference when doing the exercises and implied that the supervision of the patients was very different and dependent on the setting. Two main objectives were pursued in all the studies: to promote a physical activity target for the patients or an exercise program complying with the pulmonary rehabilitation guidelines. Only a few studies seemed to perform an exercise program closely complying with the expected one in a classical pulmonary rehabilitation, and included a follow-up after the intervention.

About the studies compared to usual cares, the length of the interventions was highly variable, from 4 weeks to 4 months and even 12 months for a study assessing the impact of a maintenance program. Again, the frequency of the sessions was often different between IG and CG. Moreover, when the duration of each session was mentioned, it was sometimes under the recommended duration.

Regarding the studies comparing the telerehabilitation to a classical or adapted pulmonary rehabilitation, the most frequent length of the programs was 3 months. Two studies assessed a shorter program. Some studies did not compare a similar frequency of sessions in the two groups. The duration of the sessions was relatively short and mainly around 30 min.

### 3.5. Quality of Studies

The quality assessment of the studies is shown in Table 2. Five studies can be considered as with a good internal validity [10,12,14,16,17]. The PEDro scores varied from 0 to 8 with a median score of 5.5. The “0” was due to the lack of eligibility criteria [10].

### 3.6. Outcome Measures

Three kinds of outcomes were investigated in this systematic review. They are presented in the Table 3.

To assess the functional exercise capacity, the six minutes walking test (6MWT) was used in the majority of the studies. Studies also included incremental or endurance shuttle walk tests, or a sit-to-stand test. The training time and an accelerometer were used without 6MWT in one study, respectively.

The dyspnoea was only assessed in ten studies and the evaluation was classical (modified Medical Research Council (mMRC), Borg scales, CRQ-D). Another study included the dyspnoea assessment in the BODE index without isolated data about dyspnoea.

Health-related quality of life and psychogenic outcomes were largely assessed in the retrieved studies. Different questionnaires were used by the authors even if the COPD assessment test (CAT), the Saint-Georges Respiratory questionnaire (SGRQ) and the Hospital Anxiety and Depression (HADS) scale were mainly found.

### 3.7. Effects of Intervention

The results of the studies were divided based on the type of CG. The effect was considered as positive when there was an inter-group difference when IG was compared to usual cares, whereas the lack of inter-group difference was considered as positive for comparisons with a classical PR.

#### 3.7.1. Effects on Functional Exercise Capacity

Regarding the functional exercise capacity, all the interventions showed positive results when compared to PR. Indeed, no difference was observed with the control group for 6MWT. When the comparison was performed with usual care, there was a benefit for all studies even if one of these demonstrated only a benefit in endurance shuttle walk test but not in the 6MWT [16].

#### 3.7.2. Effects on Dyspnoea

The dyspnoea was mainly assessed by the mMRC scale. No difference was observed neither for the comparisons to the usual care nor to another intervention. The clinical significance on the Borg scale was reached in the study comparing a telerehabilitation to the usual care [16] and only in the IG in the study comparing an exergam to a videogame in patients with IPF [18].

#### 3.7.3. Effects on Quality of Life

No positive effect was observed on the quality of life when the intervention was compared to usual care. However, one of these studies assessed a maintenance program after a pulmonary rehabilitation in patients with COPD [20] and another one evaluated the effect of very short sessions (<20 min) in patients with IPF [13]. However, one study succeeded in demonstrating a significant difference in favour of telerehabilitation when compared to exercises on video performed as a maintenance program for 12 months [17]. The anxiety and depression and the impact of COPD on personal life were systematically improved by the intervention compared to usual care.

The effects on quality of life, anxiety and depression, and the impact of COPD on personal life were similar in all the studies comparing intervention to pulmonary rehabilitation and even better on anxiety and depression, and the impact of COPD on personal life in one study [14]. This last study failed to demonstrate a maintained difference after 1 year of follow-up.

### 3.8. Adherence and Adverse Events Related to Telerehabilitation

The adherence reported in the studies ranged from 39% to 93% and always higher than 80% when a supervision during the exercise was included (Table 2). Neither diaries nor phone calls guaranteed good adherence. One study comparing two similar groups with and without interactive app highlighted a large difference in attendance in favour of the interactive app and then the supervision [9].

No study reported serious adverse events even if all except one clearly mentioned the specific assessment of the eventual adverse events. The only observed adverse event was pain in two studies [12,19]. In one of these, the number of adverse events was even smaller (2 vs. 3) than in the PR-control group.

## 4. Discussion

This systematic review aimed to summarise the different telerehabilitation programs related to a chronic respiratory disease. It highlighted positive effects on functional exercise capacity, quality of life, anxiety and depression, and impact of COPD on personal life but not on dyspnoea in COPD patients. Although, the investigated forms of telerehabilitation were often not in agreement with the guidelines about pulmonary rehabilitation.

Initially, telerehabilitation has been developed to counteract the burden of the rehabilitation, mainly the distance from home to the rehabilitation centre and the transportation difficulties for the patients. This modality was proposed as an initial rehabilitation or as a prolongation of an ongoing rehabilitation. This modality of treatment was widely developed in cancer [21] and recently expanded to many other medical conditions such as chronic lung disease [22]. With the large access to the new technologies and the unmet cares related to the lockdown and restrictions associated with the COVID-19 pandemic [22], the interest for telerehabilitation has been recently increasing in many chronic diseases.

In 2021, the authors of an American Thoracic society workshop about the future of the pulmonary rehabilitation reported that the concepts of “access,” “uptake,” and “completion” are key to the challenges facing pulmonary rehabilitation programs and they mentioned that telerehabilitation could improve accessibility, and completion of pulmonary rehabilitation programs [23]. However, these authors specified that “adoption of alternative models for pulmonary rehabilitation will require a demonstration of comparable or greater clinical outcomes to those of traditional pulmonary rehabilitation programs, as well as evaluation of safety and cost-effectiveness, staff training and guideline development.”.

The different telerehabilitation programs improved quality of life, and anxiety and depression. Similar benefits were previously demonstrated in line with telemedicine [24] even if the studied forms of telemedicine did not include exercise programs. Then it is difficult to conclude about the real benefit associated to the exercise guided by a telehealth modality or to the maintained contact by the same telehealth modality in our systematic review. Indeed, a simple phone-based intervention was previously demonstrated as beneficial on quality of life in lung cancer [24]. Moreover, the only study comparing a similar telerehabilitation tool with or without interaction with a healthcare worker demonstrated no benefit related to the supervision although the attendance was improved due to the interaction [9].

Surprisingly, the dyspnoea was not improved after telerehabilitation. It can be explained by the assessment tool used in studies (the MMRC dyspnoea scale). On the one hand, the MMRC dyspnoea scale is not very sensitive, and this scale is rather used to assess COPD severity [25]. On the other hand, the dyspnoea should be assessed by scales assessing the sensory, emotional, and impact domains [26]. Such assessments were missing in the telerehabilitation programs.

The patients with COPD were the main population of patients with a chronic respiratory disease investigated in the telerehabilitation field. This is not surprising due to the high prevalence of this disease and the highly demonstrated place of pulmonary rehabilitation in the care of this disease compared to the other chronic lung disease. Moreover, these patients are particularly good candidates to the telerehabilitation due to their difficulties related to transportation [27]. Based on the results of this systematic review, the place of telerehabilitation can be affirmed in this population due to the good adherence rate and poor number of side effects. Indeed, the adherence in the intervention groups was similar to the control groups in all the comparative studies retrieved in this systematic review and to the data found in other conditions for classical rehabilitation programs [28]. Even, if the data are sparse, they suggest that the supervision is an important criterion to guarantee a high adherence to the telerehabilitation. However, non-inclusion factors as poor motivation, frequent exacerbations, and cognitive disorders, were common in the studies suggesting the selection of motivated patients. That can contribute to a higher adherence than in the reality.

Maintenance strategies are required [29] because the well-known benefits of pulmonary rehabilitation in chronic lung diseases [1] are often difficult to maintain over the subsequent 12 months. This was confirmed by Kwon et al. who demonstrated a disappearance of the effect initially observed after the program [9]. The two studies investigating such a maintenance program with the use of telemedicine showed opposite results. Vasilopoulou et al. demonstrated a similar benefit on functional exercise capacity, QoL, and symptoms as a classical pulmonary rehabilitation and these effects were higher than usual care [17]. Even if Galdiz et al. failed to demonstrate such a benefit, it has to be noted that the evolution of the outcomes was more stable with the telerehabilitation maintenance program than with usual cares [20]. It means that even if some patients did not benefit from this program, it is interesting for some patients.

Unfortunately, this systematic review highlighted that the telerehabilitation has not yet been studied enough in the chronic lung diseases. Concluding on the effectiveness is then difficult. Moreover, we observed that some studies did not investigate a pulmonary rehabilitation that follows the international standards transformed into a telemodality with or without supervision, but only remote exercises. For example, the duration of the session was frequently shorter than 30 min which are under the recommended duration.

Some limitations related to this systematic review have to be addressed. First, the studies related to other diseases than COPD are sparse and extrapolation of the results to these diseases is not possible. Second, there is not enough studies about a specific pulmonary telerehabilitation and the programs showed a great disparity in the settings. This explains why we did not perform a meta-analysis. The choice of not-considering a phone call without other interventions as telerehabilitation can be questionable but the aim was to avoid the studies assessing home-based program without the use of new technologies. Home-based programs were previously studied.

In conclusion, this review demonstrated that the telerehabilitation has to be considered as one option of classical pulmonary rehabilitation to improve the functional exercise capacity, quality of life, anxiety and depression, and the impact of COPD on a person’s life. Until now, this conclusion cannot be extrapolated to the other chronic lung diseases due to the lack of data. As a perspective, a definition of the acceptable forms of telerehabilitation should be provided and a particular attention to the compliance of the telerehabilitation should be paid with the international guidelines on pulmonary rehabilitation. Moreover, there are missing studies about the combination between classical and tele- pulmonary rehabilitation.

## Figures and Tables

**Table 1:** Study design and participant characteristics.

Authors, Year	Disease (s)	Group	Sample (*n*)	Age (Year)	Gender (M/F)	FEV1 (%)	BMI (kg/m^2^)	Drop-Out
Tabak 2014 [15]	COPD	IG	14	65.2 ± 9.0	8/6	48.7 ± 16.7	28.4 ± 7.8	0
		CG	16	67.9 ± 5.7	11/5	56.4 ± 10.6	29.2 ± 4.7	0
Chaplin 2017 [11]	COPD	IG	51	66.4 ± 10.1	38/14	58.7 ± 29.1	27.9 ± 6.4	29
		CG	52	66.1 ± 8.1	33/19	55.0 ± 20.5	29.3 ± 6.3	12
Franke 2016 [19]	COPD	IG-CG	23	63.3 ± 7.8	20/24	47.5 ± 15.8	24.3 ± 5.2	5
		CG-IG	21					2
Bourne 2017 [12]	COPD	IG	64	69.1 ± 7.9	18/8	58.0 ± 23.6	NR	7
		CG	26	71.4 ± 8.6	41/23	60.5 ± 20.1	NR	5
Tsai 2017 [16]	COPD	IG	20	73 ± 8	12/7	60 ± 23	28 ± 4	1 (unrelated)
		CG	17	75 ± 9	6/11	68 ± 19	28 ± 5	0
Vasilopoulou 2017 [17]	COPD	IG	47	66.9 ± 9.6	44/3	49.6 ± 21.9	28.0 ± 5.3	0
		CG1	50	66.7 ± 7.3	38/12	51.8 ± 17.3	27.5 ± 5.0	0
		CG2	50	64.0 ± 8.0	37/13	51.7 ± 21.0	26.4 ± 5.0	0
Bernocchi 2018 [8]	COPD + CHF	IG	56	71 ± 9	50/6	66.6 ± 18.6	28.5 ± 5.8	11
		CG	56	70 ± 9.5	42/14	66.1 ± 16.4	27.7 ± 5.4	21
Kwon 2018 [9]	COPD	IG1	27	64 ± 8	23/4	58.6 ± 15.8	23.6 ± 3.7	11
		IG2	30	65 ± 7	26/4	57.1 ± 16.8	22.6 ± 3.0	6
		CG	28	64 ± 8	21/7	55.8 ± 15.5	24.3 ± 3.9	6
Knox 2019 [10]	COPD + CLD	IG	21	70.1 ± 10.8	14/7	NR	NR	0
		CG	24	68.6 ± 12.8	10/14	NR	NR	1
Yuen 2019 [18]	IPF	IG	10	67.4 ± 7.4	5/5	65 ± 15	28.0 ± 4.6	3
		CG	10	72.2 ± 8.4	8/2	86 ± 18	28.4 ± 4.3	0
Hansen 2020 [14]	COPD	IG	67	68.4 ± 8.7	32/35	32.6 ± 10.3	25.5 ± 5.0	10
		CG	67	68.2 ± 9.4	28/39	33.7 ± 8.4	25.9 ± 6.4	14
Galdiz 2021 [20]	COPD	IG	41	63.0 ± 6.6	27/14	42.1 ± 14.6	26.5 ± 5.2	2
		CG	40	62.3 ± 8.2	27/13	45.9 ± 17.2	27.7 ± 5.1	2
Cerdan 2021 [13]	IPF	IG	15	70.1 ± 8.8	13/2	NR	NR	5
		CG	14	72.4 ± 7.6	8/6	NR	NR	3

Age and BMI values are presented as mean ± SD or median (IQR). BMI: body mass index; IG: intervention group; CG: control group; CHF: chronic heart failure; IPF: idiopathic pulmonary fibrosis; CLD: chronic lung disease; NR: not reported.

**Table 2:** Intervention characteristics and quality of studies compared to usual care (**A**) or to PR (**B**).

Authors, Year	Group	Setting, Supervision	Length of Intervention	Frequency Duration	Exercise Intervention	Follow-Up	Adherence (A) Adverse Events (AE)	PEDro
(**A**) Studies using usual care as control group								
Tabak 2014 [15]	IG	Accelerometer Smartphone Web diary No	4 w	>4/w	Feedback text messages (awareness and extra motivation) based on the difference between the activity and the reference Advice on how to improve or maintain the activity behaviour	-	A: 54% AE: NR	5
	CG	UC	4 w		Medication and physiotherapy (training session)	-	A: NR AE: NR	
Tsai 2017 [16]	IG	Videoconference Lower limb cycloergometer Oximeter Yes	8 w	3/w 55 min	Lower limb cycle ergometer, walking training and strengthening exercises	-	A: 92% AE: no	8
	CG	UC			Optimal pharmacological intervention Action plan	-	A: NR	
Vasilopoulou 2017 [17]	IG	Video Psychological support Weekly contact Multimodal apparatus (pedometer) Tablet Call centre No	2 m PR + 12 m Maintenance TelePR (144 sessions) No	3/w NR	Exercise (arm and leg exercises and walking drills) Action plan	-	A: 93% AE: no	5
	CG1	PR Yes	2 m PR + 12 m PR (96 sessions)	2/w NR	Exercise	-	A: 91% AE: no	
	CG2	UC No	2 m PR + 12 m UC		Optimal pharmacological treatment	-	A: NR	
Kwon 2018 [9]	IG1	TelePR Non-interactive App Monitoring website Oximeter (connected) No	12 w (fixed)		Walking (fixed program) (6 levels of distance, automatic increment)	-	A: 59% AE: NR	3
	IG2	TelePR Interactive App Monitoring website Oximeter (connected) Yes	6 w (fixed) + 6 w (Interactive)		Walking (interactive program) (12 levels and automatic adaptation based on the Borg scale)	-	A: 80% AE: NR	
	CG	UC				-	A: 79%	
Bernocchi 2018 [8]	IG	TelePR kit (ergometer, pedometer) Phone call Oximeter + ECG No	4 m No	From 3/w + 2/w to 3 to 7/w From 45–55 min to 30 to 45 min	Initial level Ergometer without load (3/w) Walking (2/w) Final level Ergometer (from 0 to 60 W) Muscle reinforcement (0.5 kg) Pedometer	2 m	A: <60% AE: no	4
	CG	UC			Pharmacological treatment PTE	2 m		
Galdiz 2021 [20]	IG	TelePR kit (phone, oximeter) No	8 w PR + 12 m Maintenance TelePR	3/w 60 min	30 min weightlifting 30 min on cycle ergometer 4 sessions of PTE	3, 9, 12 m	A: 60% AE:no	7
	CG	UC No	8 w PR + 12 m UC		Advice: 1 h walking/d	3, 9, 12 m	A: NR AE: NR	
Cerdan 2021 [13]	IG	TelePR Video Chat Oximeter Tablet No	12 w	3–5/w 10–20 min	Exercise E-learning	3, 6 m	A: 64% AE: NR	
	CG	UC No	12 w		Pharmacological treatment			
(**B**) Studies using exercise or PR as control group								
Chaplin 2017 [11]	IG	TelePR Weekly phone call No	6/8 w	Target weekly work	Exercise (aerobic and upper/lower limb resistance training) PTE	-	A: NR AE: 20%	5
	CG	PR Yes	7 w	2/w 120 min	Exercise (aerobic and upper/lower limb resistance training) (60 min) PTE (60 min)	-	A: NR AE: 5%	
Franke 2016 [19]	IG	Ergometer with data transmission Phone call No	3 m	7/w 30 min	Ergometer with motivational phone call every week	-	A: 69% AE: NR	4
	CG	Ergometer No	3 m	7/w 30 min	Ergometer without motivational phone call		A: 61% AE: NR	
Bourne 2017 [12]	IG	TelePR No	6 w	2 to 5/w 10 min to 35 min	10 exercises (Biceps curls, squats, push-ups against a wall, leg extensions in a sitting position, upright row with weights, sit-to-stand, arm swings with a stick, leg kicks to the side, arm punches with weights and step-ups) PTE	-	A: 62% AE: 2	8
	CG	PR Yes	6 w	2/w (PR) 3/w (home) NR	10 exercises (Biceps curls, squats, push-ups against a wall, leg extensions in a sitting position, upright row with weights, sit-to-stand, arm swings with a stick, leg kicks to the side, arm punches with weights and step-ups. PTE	-	A: 72% AE: 3	
Vasilopoulou 2017 [17]	IG	Video Psychological support Weekly contact Multimodal apparatus (pedometer) Tablet Call centre No	2 m PR + 12 m Maintenance TelePR (144 sessions) No	3/w NR	Exercise (arm and leg exercises, and walking drills) Action plan	-	A: 93% AE: no	5
	CG1	PR Yes	2 m PR + 12 m PR (96 sessions)	2/w NR	Exercise	-	A: 91% AE: no	
	CG2	UC No	2 m PR + 12 m UC		Optimal pharmacological treatment	-	A: NR	
Knox 2019 [10]	IG	TelePR Videoconference Yes	7 w	2/w 60–90 min + 20–40 min (spoke site)	Exercise PTE	-	A: 61.9% AE: 0	0
	CG	PR Yes	7 w	2/w 60–90 min + 20–40 min (hub site)	Exercise PTE	-	A: 54.6% AE: 1	
Yuen 2019 [18]	IG	Wii U console Balance Board Wii Fit game Phone call No	12 w	3/w 30 min	Exergame	-	A: 39% AE: 0	7
	CG	Nintendo Wii U console Traditional video game Phone call No	12 w	3/w 30 min	Video game	-	A: NR AE: 0	
Hansen 2020 [14]	IG	TelePR Videoconference Yes	10 w	3/w 35 min	High repetitive muscle exercise PTE	3, 12 m	A: 83% AE: 2	7
	CG	PR Yes	10 w	2/w 60 min	Exercise PTE		A: 80% AE: 0	

IG: intervention group; CG: control group; UC: usual care; w; week; A: adherence; AE: adverse events; PR: pulmonary rehabilitation; m: month; NR: non reported; PTE: patient therapeutic education.

**Table 3:** Results of the outcomes retrieved from the included studies. The studies are divided in two categories based on the comparators used in the control group.

Authors, Year	Functional Exercise Capacity	Dyspnoea	HRQoL Anxiety-Depression
Studies using usual care as control group			
Tabak 2014 [15]	Accelerometer IG: 5766 ± 965–6106 ± 965–6271 ± 959–5603 ± 964 CG: 5256 ± 865–4853 ± 865–4590 ± 865–4617 ± 865 *p* = 0.482	MRC IG: 2.0 ± 0.9 (Δ: −0.3 ± 0.7) CG: 2.3 ± 1.4 (Δ: −0.2 ± 0.9)	CCQ IG: 2.0 ± 0.8 (Δ: −0.3 ± 0.5) CG: 1.8 ± 1.0 (Δ: 0.0 ± 0.6)
Tsai 2017 [16]	6MWT (m) IG: 363 (66)–403 (82) (+40 (1/80)) CG: 383 (93)–374 (136) (−9 (−62/44) IG vs. CG: 45 (−18/108) ISWT (m) IG: 260 (106)–275 (132) (+12 (−12/36)) CG: 298 (114)–306 (118) (+8 (−8/24)) IG vs. CG: 6 (−23/35) ESWT (s) IG: 410 (253)–693 (357) (283 (107/460)) CG: 361 (155)–316 (182) (−31 (−76/14)) IG vs. CG: 340 (153/526)	Borg IG: 4 (2)–3 (2) (+1 (0/2)) CG: 4 (2)–4 (2) (0 (−1/1)) IG vs. CG: +4 (−7/16)	CRDQ IG: 90 (18)–99 (16) (+9 (2/16) CG: 88 (23)–90 (18) (+2 (−5/10) IG vs. CG: +8 (−1/16) CAT IG: 16 (7)–15 (7) (−1 (−4/2) CG: 15 (6)–18 (6) (+3 (1/5) IG vs. CG: −3 (−7/0) HADS-A IG: 5 (4)–4 (4) (−2 (−3/−0.3) CG: 6 (4)–6 (3) (−1 (−2/1) IG vs. CG: −1 (−3/0) HADS-D IG: 5 (3)–4 (3) (−1.4 (−2/−0.4) CG: 5 (3)–6 (3) (+1(0/3)) IG vs. CG: −3 (−4/−1)
Vasilopoulou 2017 [17]	6MWT (m) IG: 389.1 ± 91.3–422.1 ± 70.5–420.2 ± 74.9 (NS) CG1: 385.1 ± 80.3–423.0 ± 70.5–427.5 ± 63.0 (NS) CG2: 384.8 ± 80.2–382.4 ± 80.3–339.9 ± 110.1 (NS) IG et CG1 vs. CG2: S	mMRC IG: 1.8 ± 0.9–1.6 ± 1.0 CG1: 1.5 ± 0.9–1.3 ± 0.9 CG2: 2.5 ± 1.0–3.1 ± 0.8 IG vs. CG1 vs. CG2: S	SGRQ IG: 42.2 ± 19.2–38.4 ± 20.5 CG1: 35.4 ± 15.7–33.6 ± 16.5 CG2: 44.7 ± 16.9–50.2 ± 17.7 IG vs. CG1 vs. CG2: S CAT IG: 12.9 ± 7.5–13.0 ± 7.3 CG: 13.2 ± 5.8–11.8 ± 5.6 CG2: 16.1 ± 6.2–20.9 ± 6.7 IG vs. CG1 vs. CG2: S
Kwon 2018 [9]	6MWT (m) IG1: NS IG2: NS CG: NS	MRC IG1: NS IG2: NS CG: NS	CAT IG1: *p* = 0.01 (6 w), NS (6 w) IG2: *p* = 0.06 (6 w), NS (6 w)
Bernocchi 2018 [8]	6MWT (m) IG: +60 (22.2/97.8) CG: −15 (−40.3/9.8) IG vs. CG: *p* = 0.004	MRC IG: −0.17 (−0.3/−0.02) CG: 0.07 (−0.1/0.3) IG vs. CG: *p* = 0.05	CAT IG: −5.3 (−6.9/−3.7) CG: +1.6 (−0.4/3.5) IG vs. CG: *p* < 0.001
Galdiz 2021 [20]	6MWT (m) IG: 445 m (102)–436 (113)–432 (117)–441 (106) CG: 449 m (92)–447 (95)–423 (117)–423 (101) IG vs. CG: 19.9 m (−4.1/43.8)(*p* = 0.10)	BODE IG vs. CG: 0.0 (−0.6/0.6) (*p* = 0.89) CRQ-D IG: 5.2 (1.2)–5.2 (1.1)–4.9 (1.2)–4.8 (1.5) CG: 5.2 (1.5)–4.7 (1.5)–4.8 (1.3)–5.0 (1.3) IG vs. CG: NR	CRQ-F IG: 4.7 (1.1)–4.7 (1.0)–4.5 (1.2)–4.5 (1.3) CG: 4.8 (1.3)–4.2 (1.4)–4.1 (1.2)–4.3 (1.5) IG vs. CG: +0.3 (−0.2/0.8) (*p* = 0.20) CRQ-E IG: 5.1 (1.2)–5.3 (1.0)–5.1 (1.2)–5.1 (1.2) CG: 5.2 (1.3)–4.6 (1.4)–4.7 (1.3)–4.8 (1.3 IG vs. CG: +0.4 (0.0/0.8) (*p* = 0.067) CRQ-M IG: 5.2 (1.5)–5.5 (1.2)–5.2 (1.5)–5.1 (1.4) CG: 5.3 (1.5)–4.9 (1.5)–5.0 (1.5)–5.0 (1.5) IG vs. CG: +0.2 (−0.4/0.7) (*p* = 0.55)
Cerdan 2021 [13]	6MWT (m) IG: 461.5 ± 115–470 ± 115 (S)–469 ± 136 (S)–448 ± 133 CG: 446 ± 63.6–421 ± 70–423 ± 76–390 ± 85 IG vs. CG: NR–+39.5 (*p* = 0.03)–+34.3 (*p* = 0.02)–+40.0 (*p* = 0.15).		SGRQ-I IG: 49.8 ± 14.9–51.2 ± 17.8–48.3 ± 13.3–43.9 ± 19.4 CG: 47.7 ± 16.7–43.3 ± 16.4–49.7 ± 22.2–45.9 ± 16.6 IG vs. CG: NS GAD7 IG: 1.63 ± 2.5–3.27 ± 3.9–2.9 ± 3.1–2.1 ± 3.2 CG: 2.36 ± 3.9–2.55 ± 3.3–0.8 ± 1.7–4.6 ± 3.7 IG vs. CG: NS
Studies using exercise or PR as control group			
Chaplin 2017 [11]	ESWT (s) IG: +189 ± 211.1 CG: +184.5 ± 247.4 IG vs. CG: NS	CRQ-D IG: +0.7 ± 1.2 CG: +0.8 ± 1.0 IG vs. CG: NS	CAT: NR (NS) HADS: NR (NS)
Franke 2016 [19]	Training time (min) IG: 24.2 ± 9.4 CG: 19.6 ± 10.3 IG vs. CG: *p* < 0.001 IGt1: 26.7 ± 8.4 IGt2: 21.5 ± 9.9 IGt1 vs. IGt2: *p* = 0.066		CAT IG: 15.3 ± 7.6 (*p* = 0.006) CG: 15.7 ± 7.3 (*p* = 0.03) IG vs. CG: *p* = 0.03
Bourne 2017 [12]	6MWT (m) IG: 416.5 ± 118.3–445.1 ± 124.9 CG: 388.7 ± 104.4–433.6 ± 102.9 IG vs. CG: *p* = 0.10	mMRC IG: 2.0 (1-2)–1.5 (1-2) CG: 2 (1-3)–1 (1-2) IG vs. CG: *p* = 0.91	SGRQ IG: 37.7 ± 17.2–39.3 ± 18.5 CG: 42.4 ± 18.6–39.3 ± 18.5 IG vs. CG: *p* = 0.30 CAT IG: 17.3 ± 6.7–16.2 ± 6.7 CG: 18.1 ± 7.9–14.9 ± 7.0 IG vs. CG: *p* = 0.37 HADS IG: 10.0 (6-18)–10.5 (5-13) CG: 10.0 (6-16.5)–7 (4-15) IG vs. CG: *p* = 0.26
Vasilopoulou 2017 [17]	6MWT (m) IG: 389.1 ± 91.3–422.1 ± 70.5–420.2 ± 74.9 (NS) CG1: 385.1 ± 80.3–423.0 ± 70.5–427.5 ± 63.0 (NS) CG2: 384.8 ± 80.2–382.4 ± 80.3–339.9 ± 110.1 (NS) IG et CG1 vs. CG2: S	mMRC IG: 1.8 ± 0.9–1.6 ± 1.0 CG1: 1.5 ± 0.9–1.3 ± 0.9 CG2: 2.5 ± 1.0–3.1 ± 0.8 IG vs. CG1 vs. CG2: NR	SGRQ IG: 42.2 ± 19.2–38.4 ± 20.5 CG1: 35.4 ± 15.7–33.6 ± 16.5 CG2: 44.7 ± 16.9–50.2 ± 17.7 IG vs. CG1 vs. CG2: NR CAT IG: 12.9 ± 7.5–13.0 ± 7.3 CG: 13.2 ± 5.8–11.8 ± 5.6 CG2: 16.1 ± 6.2–20.9 ± 6.7 IG vs. CG1 vs. CG2: NR
Knox 2019 [10]	ISWT (m) IG: +137 (S) CG: +66 (S) IG vs. CG: *p* = 0.025	MRC IG: 3.3 ± 0.8–−0.75 ± 0.86 (*p* = 0.003) CG: 3.5 ± 0.9–−0.48 ± 0.60 (*p* = 0.002) IG vs. CG: *p* = 0.26	CAT IG: 24.0 ± 6.2 (S) CG: 25.2 ± 6.6 (S) IG vs. CG: *p* = 0.51 HADS-A IG: 7.6 ± 4.2 (S) CG: 8.2 ± 3.5 IG vs. CG: *p* = 0.18 HADS-D IG: 7.05 ± 2.60 CG: 6.50 ± 2.70 IG vs. CG: *p* = 0.07
Yuen 2019 [18]	6MWT (m) IG: 321 ± 88–305 ± 108 (*p* = 0.27) CG: 408 ± 103–372 ± 125 (*p* = 0.17) IG vs. CG: *p* = 0.29	Borg IG: 2.4 ± 1.3–3.4 ± 1.6 (*p* = 0.02) CG: 1.65 ± 1.3–1.4 ± 1.2 (*p* = 0.54) IG vs. CG: *p* = 0.99	SGRQ IG: 44 ± 12–47 ± 15 (*p* = 0.30) CG: 32 ± 10–33 ± 17 (*p* = 0.70) IG vs. CG: *p* = 0.63
Hansen 2020 [14]	6MWT (m) IG: 17.2 (5.8/28.5)(S) − 22.0 (5.0/39.1)(S) CG: 23.5 (12.1/35.0)(S) − 11.0 (−5.2/27.2) IG vs. CG (end–12 m): 8.3 (−7.7/24.3) − −3.9 (−27.9/19.9) 30sSTST IG: 1.3 (0.4/2.0)(S)–1.1 (0.1/2.0)(S) CG: 1.7 (0.9/2.5)(S)–1.5 (0.5/2.3)(S) IG vs. CG (end–12 m): 0.5 (−0.6/1.5)–0.5 (−0.6/1.6)		CCQ IG: −0.3 (−0.4/−0.1)(S)−0.0 (−0.2/0.2) CG: −0.1 (−0.3/0.1)−0.1 (−0.2/0.3) IG vs. CG (end–12 m): 0.2 (−0.1/0.5) − 0.1 (−0.1/0.4) EQ5D-VAS IG: 3.2 (−1.2/7.6)−3.5 (−1.2/8.2) CG: 2.9 (−1.4/7.2)−4.2 (−0.4/9.0) IG vs. CG (end–12 m): −0.2 (−6.2/5.9) − 1.6 (−5.1/8.3) CAT IG: −1.7 (−3.2/−0.2)(S)–−0.5 (−1.9/1.1) CG: −0.3 (−1.8/1.2)–−1.0 (−2.5/0.6) IG vs. CG (end–12 m): 1.6 (0.1/3.3)(S)–−0.2 (−2.1/1.8) HADS-A IG: −1.0 (−1.7/−0.2)(S)–−0.5 (−1.4/0.5) CG: 0.1 (−0.6/0.8)–−0.3 (−1.2/0.7) IG vs. CG (end–12 m): 1.2 (0.2/2.3)(S)−0.4 (−0.8/1.6) HADS-D IG: −0.4 (−1.1/0.3)−0.5 (−0.4/1.5) CG: 0.3 (−0.4/1.0)−0.3 (−0.6/1.4) IG vs. CG (end–12 m): 0.9 (0.1/1.7)(S) –−0.2 (−1.3/1.0)

Data are presented as mean ± SD or median (IQR) IG: intervention group; CG: control group; MRC: Medical Research Council; Δ: difference; 6MWT: six minutes walking test; ISWT: incremental shuttle walk test; ESWT: endurance shuttle walk test; CCQ: Clinical COPD Questionnaire; CRDQ: Chronic Respiratory Disease Questionnaire; CAT: COPD assessment test; HADS-A: Hospital Anxiety and Depression scale–domain anxiety; HADS-D: Hospital Anxiety and Depression scale–domain depression; SGRQ: Saint-Georges Respiratory Questionnaire; CRQ: Chronic Respiratory Questionnaire; GAD7: Generalised Anxiety Disorder; EQ5D-VAS: EuroQoL Five dimensions.

## Data Availability

Data are available on request.

## References

[1] Spruit M.A., Singh S.J., Garvey C., ZuWallack R., Nici L., Rochester C., Hill K., Holland A.E., Lareau S.C., Man W.D.C. (2013). An official American Thoracic Society/European Respiratory Society statement: Key concepts and advances in pulmonary rehabilitation. Am. J. Respir. Crit. Care Med..

[2] Wuytack F., Devane D., Stovold E., McDonnell M., Casey M., McDonnell T.J., Gillespie P., Raymakers A., Lacasse Y., McCarthy B. (2018). Comparison of outpatient and home-based exercise training programmes for COPD: A systematic review and meta-analysis. Respirology.

[3] Cox N.S., Oliveira C.C., Lahham A., Holland A.E. (2017). Pulmonary rehabilitation referral and participation are commonly influenced by environment, knowledge, and beliefs about consequences: A systematic review using the Theoretical Domains Framework. J. Physiother..

[4] Augustine A., Bhat A., Vaishali K., Magazine R. (2021). Barriers to pulmonary rehabilitation—A narrative review and perspectives from a few stakeholders. Lung.

[5] Sami R., Salehi K., Hashemi M., Atashi V. (2021). Exploring the barriers to pulmonary rehabilitation for patients with chronic obstructive pulmonary disease: A qualitative study. BMC Health Serv. Res..

[6] Ozden F., Sari Z., Karaman O.N., Aydogmus H. (2021). The effect of video exercise-based telerehabilitation on clinical outcomes, expectation, satisfaction, and motivation in patients with chronic low back pain. Ir. J. Med. Sci..

[7] Moher D., Liberati A., Tetzlaff J., Altman D.G. (2009). Preferred reporting items for systematic reviews and meta-analyses: The PRISMA statement. J. Clin. Epidemiol..

[8] Bernocchi P., Vitacca M., La Rovere M.T., Volterrani M., Galli T., Baratti D., Paneroni M., Campolongo G., Sposato B., Scalvini S. (2018). Home-based telerehabilitation in older patients with chronic obstructive pulmonary disease and heart failure: A randomised controlled trial. Age Ageing.

[9] Kwon H., Lee S., Jung E.J., Kim S., Lee J.K., Kim D.K., Kim T.H., Lee S.H., Lee M.K., Song S. (2018). An mHealth Management Platform for Patients with Chronic Obstructive Pulmonary Disease (efil breath): Randomized Controlled Trial. JMIR mHealth uHealth.

[10] Knox L., Dunning M., Davies C.A., Mills-Bennet R., Sion T.W., Phipps K., Stevenson V., Hurlin C., Lewis K. (2019). Safety, feasibility, and effectiveness of virtual pulmonary rehabilitation in the real world. Int. J. Chron. Obstruct. Pulmon. Dis..

[11] Chaplin E., Hewitt S., Apps L., Bankart J., Pulikottil-Jacob R., Boyce S., Morgan M., Williams J., Singh S. (2017). Interactive web-based pulmonary rehabilitation programme: A randomised controlled feasibility trial. BMJ Open.

[12] Bourne S., DeVos R., North M., Chauhan A., Green B., Brown T., Cornelius V., Wilkinson T. (2017). Online versus face-to-face pulmonary rehabilitation for patients with chronic obstructive pulmonary disease: Randomised controlled trial. BMJ Open.

[13] Cerdán-de-Las-Heras J., Balbino F., Løkke A., Catalán-Matamoros D., Hilberg O., Bendstrup E. (2021). Tele-Rehabilitation Program in Idiopathic Pulmonary Fibrosis-A Single-Center Randomized Trial. Int. J. Environ. Res. Public Health.

[14] Hansen H., Bieler T., Beyer N., Kallemose T., Wilcke J.T., Østergaard L.M., Andeassen H.F., Martinez G., Lavesen M., Frølich A. (2020). Supervised pulmonary tele-rehabilitation versus pulmonary rehabilitation in severe COPD: A randomised multicentre trial. Thorax.

[15] Tabak M., Vollenbroek-Hutten M.M., van der Valk P.D., van der Palen J., Hermens H.J. (2014). A telerehabilitation intervention for patients with Chronic Obstructive Pulmonary Disease: A randomized controlled pilot trial. Clin. Rehabil..

[16] Tsai L.L., McNamara R.J., Moddel C., Alison J.A., McKenzie D.K., McKeough Z.J. (2017). Home-based telerehabilitation via real-time videoconferencing improves endurance exercise capacity in patients with COPD: The randomized controlled TeleR Study. Respirology.

[17] Vasilopoulou M., Papaioannou A.I., Kaltsakas G., Louvaris Z., Chynkiamis N., Spetsioti S., Kortianou E., Genimata S.A., Palamidas A., Kostikas K. (2017). Home-based maintenance tele-rehabilitation reduces the risk for acute exacerbations of COPD, hospitalisations and emergency department visits. Eur. Respir. J..

[18] Yuen H.K., Lowman J.D., Oster R.A., de Andrade J.A. (2019). Home-Based Pulmonary Rehabilitation for Patients with Idiopathic Pulmonary Fibrosis: A Pilot Study. J. Cardiopulm. Rehabil. Prev..

[19] Franke K.J., Domanski U., Schroeder M., Jansen V., Artmann F., Weber U., Ettler R., Nilius G. (2016). Telemonitoring of home exercise cycle training in patients with COPD. Int. J. Chron. Obstruct. Pulmon. Dis..

[20] Galdiz J.B., Gómez A., Rodriguez D., Guell R., Cebollero P., Hueto J., Cejudo P., Ortega F., Sayago I., Chic S. (2021). Telerehabilitation Programme as a Maintenance Strategy for COPD Patients: A 12-Month Randomized Clinical Trial. Arch. Bronconeumol..

[21] Ibeggazene S., Turner R., Rosario D., Bourke L. (2021). Remote interventions to improve exercise behaviour in sedentary people living with and beyond cancer: A systematic review and meta-analysis. BMC Cancer.

[22] Zasadzka E., Trzmiel T., Pieczynska A., Hojan K. (2021). Modern Technologies in the Rehabilitation of Patients with Multiple Sclerosis and Their Potential Application in Times of COVID-19. Medicina.

[23] Holland A.E., Cox N.S., Houchen-Wolloff L., Rochester C.L., Garvey C., ZuWallack R., Nici L., Limberg T., Lareau S.C., Yawn B.P. (2021). Defining Modern Pulmonary Rehabilitation. An Official American Thoracic Society Workshop Report. Ann. Am. Thorac. Soc..

[24] Pang L., Liu Z., Lin S., Liu Z., Liu H., Mai Z., Liu Z., Chen C., Zhao Q. (2020). The effects of telemedicine on the quality of life of patients with lung cancer: A systematic review and meta-analysis. Ther. Adv. Chronic Dis..

[25] Bestall J.C., Paul E.A., Garrod R., Garnham R., Jones P.W., Wedzicha J.A. (1999). Usefulness of the Medical Research Council (MRC) dyspnoea scale as a measure of disability in patients with chronic obstructive pulmonary disease. Thorax.

[26] Parshall M.B., Schwartzstein R.M., Adams L., Banzett R.B., Manning H.L., Bourbeau J., Calverley P.M., Gift A.G., Harver A., Lareau S.C. (2012). An official American Thoracic Society statement: Update on the mechanisms, assessment, and management of dyspnoea. Am. J. Respir. Crit. Care Med..

[27] Keating A., Lee A.L., Holland A.E. (2011). Lack of perceived benefit and inadequate transport influence uptake and completion of pulmonary rehabilitation in people with chronic obstructive pulmonary disease: A qualitative study. J. Physiother..

[28] Piraux E., Caty G., Renard L., Vancraeynest D., Tombal B., Geets X., Reychler G. (2021). Effects of high-intensity interval training compared with resistance training in prostate cancer patients undergoing radiotherapy: A randomized controlled trial. Prostate Cancer Prostatic Dis..

[29] Spruit M.A., Singh S.J. (2013). Maintenance programs after pulmonary rehabilitation: How may we advance this field?. Chest.

